# Methamphetamine Induces Striatal Cell Death Followed by the Generation of New Cells and a Second Round of Cell Death in Mice

**DOI:** 10.2174/157015911795017281

**Published:** 2011-03

**Authors:** I. K Tulloch, L Afanador, J Zhu, J. A Angulo

**Affiliations:** Hunter College of the City University of New York, Department of Biological Sciences, New York NY, USA

**Keywords:** Methamphetamine, neurotoxicity, cytogenesis, striatum, striatal volume, normalization.

## Abstract

Our laboratory has been investigating the impact of a neurotoxic exposure to methamphetamine (METH) on cellular components of the striatum post-synaptic to the dopaminergic terminals. A systemic bolus injection of METH (30 mg/kg, ip) induces the production of new cells in the striatum during a period lasting from 24-48 hours after METH. The newly generated cells arise from dormant striatal progenitors and not from the subventricular zone. The newly generated cells display glial phenotypes and begin to die 24 hours after birth, or 2.5 days post-METH. The protracted phase of cell death lasts for at least three months post-METH at which time the bulk of the newly generated cells have disappeared. The METH-induced production of new cells is associated with enlarged striatal volume (up to 50% larger than controls in some animals). As the newly generated cells die over a period of three months, the enlarged striatal volume normalizes. In conclusion, a neurotoxic dose of METH induces the generation of new cells in the striatum associated with enlarged striatal volume. The new cells die over three months post-METH and the enlarged striatal volume returns to control levels. This observation is significant because studies involving METH users show striatal enlargement and the normalization of striatal volume in METH users who have been abstinent for up to 20 months.

## INTRODUCTION

Methamphetamine (METH) is an illicit drug that has been increasing in popularity for recreational use and according to recent estimates has become the second most abused drug after marijuana [1]. The illicit use of METH is a great burden on society because METH use is associated with aggressive behavior that is a major problem for law enforcement [2, 3]. Recent studies demonstrate that acute and chronic METH use induces neurotoxic responses in the human brain that can further deteriorate into neural damage including structural abnormalities. For example, studies utilizing positron emission tomography showed decreased levels of dopamine transporter [4, 5] and serotonin transporter sites [6] in various brain regions including the caudate and putamen. Detoxified METH users showed increased glucose metabolism (an index of brain function) in parietal brain regions but decreased glucose utilization in the striatum and thalamus [7]. Magnetic resonance imaging studies demonstrated that the brains of METH users display significant deficits in gray-matter in the cingulated, limbic and paralimbic cortices suggesting the loss of neurons [8]. A separate study demonstrated the enlargement of the caudate nucleus, globus pallidus and putamen, nucleus accumbens and the parietal cortex of adult METH users [9, 10].

The mechanism of action of METH has been studied extensively in animal model species such as the rodent. In the rodent brain, METH induces long-term depletion of dopamine transporters and tyrosine hydroxylase in striatal dopaminergic terminals [11, 12], depletion of tryptophan hydroxylase in serotonergic terminals [13], and degeneration of neural tissue in the striatum [14]. In addition to deficits in pre-synaptic dopaminergic terminals of the striatum, METH induces the loss of neurons in various brain regions such as the cortex and striatum [15-17]. In the present study, we have investigated the METH-induced loss of striatal neurons over a period of three months. METH induces rapid cell loss (24 hours) followed by the generation of new cells that die over a period of three months. The increased number of new cells is associated with enlargement of the striatum that normalizes over a period of three months post-METH.

## MATERIALS AND METHODS

### Animals

Male ICR mice (Taconic, Germantown, NY) between 10 to 13 weeks of age were housed individually on a 12-h light/dark cycle with food and water available *ad libitum*. The mice were habituated for two weeks prior to commencement of intraperitoneal (i.p.) drug administration. All procedures regarding animal use were performed in accordance with the *National Institutes of Health Guide for the Care and Use of Laboratory Animals* and were approved by the Institutional Animal Care and Use Committee at Hunter College of the City University of New York.

### Drug Preparation and Treatment

(+)-Methamphetamine hydrochloride (Sigma, St. Louis, MO) was dissolved in 10 mM phosphate-buffered saline, pH 7.4 (PBS) and injected i.p. at a dose of 30 mg/kg of body weight in a volume of 200 µL. 5-Bromo-2’-deoxyuridine (Sigma, St. Louis, MO) was dissolved in water and injected at a dose of 100 mg/kg (i.p.).

### Proliferation

After two weeks of habituation, male ICR mice were injected with 30 mg/kg METH or saline vehicle (i.p). After METH, different groups of animals were injected with 100 mg/kg of bromo-deoxyuridine at the following times: 2, 24, 36 hours, 2, 3, 4, 5, 6, 7, 14 and 21 days. To allow for a complete round of mitotic cell division, animals were sacrificed 24 hours after the bromo-deoxyuridine injection. They were anesthetized with a mixture of 100mg/kg ketamine + 3 mg/kg acepromazine, then perfused with PBS, followed by 4% paraformaldehyde in PBS. Following perfusion, the brains were removed from the skull and post-fixed in 4 % paraformaldehyde overnight at 4^o^C, then cryo-protected in 30% sucrose in PBS solution for 24 hours at 4^o^C. After cryo-protection, the brains were stored at 80^o^C until used.

### Apoptosis of Newly Generated Cells

49 Male ICR mice received a single METH injection (30mg/kg, i.p.) followed by a single 100mg/kg injection of bromo-deoxyuridine at 1.5 days post-METH. In order to quantify the percentage of the new cells displaying pyknotic nuclei, seven animals were randomly sacrificed and the tissue collected within five hours after the bromodeoxyuridine injection. Total bromodeoxyuridine incorporation into nuclei from these animals was used as baseline for comparison. The other animals were randomly sacrificed at the following post-METH time points: 2 days, 1, 2, 4, 8 or 12 weeks. Tissue was collected and processed in the same manner as in the proliferation study above.

### Immunohistochemistry

Coronal sections from one hemisphere were cut at 40 μm thickness and serially collected from the striatum between bregma 0.02 and 1.4 mm [18]. Six sections per animal were processed using the free-floating method. Fluorescent immunostaining was visualized and quantified as follows: PBS wash, followed by incubation at 65^o^C in a solution of 1:1 formamide and 0.375M sodium chloride/0.0375M sodium citrate (pH 7) for two hours, then incubation in 2N HCL at 37^o^C for 30 minutes. After a 10-minute rinse in 0.1M boric acid (pH 8.5), non-specific binding sites were blocked with 5% donkey serum in 0.2% Triton X100 in PBS at room temperature for one hour. Sections were then incubated in primary antibody, sheep anti-bromo-deoxyuridine immunoglobulin (1:500; Novus Biologicals, Littleton, CO) in 1% donkey serum overnight at 4^o^C. After two PBS washes (five minutes each), the sections were incubated for one hour in secondary antibody, donkey anti-sheep conjugated to FITC (1:500; Novus Biologicals, Littleton, CO) at room temperature. After two washes in PBS (five minutes each), the sections were mounted onto superfrost glass slides, sealed and coverslipped with Vectorshield hard set^TM^ mounting medium for fluorescence (Vector Laboratories, Burlingame CA).

### Quantification and Statistical Analysis

**Fig. (1):**
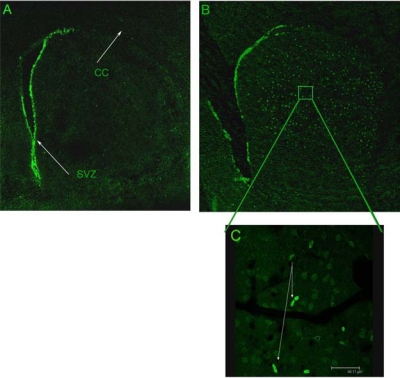
METH induces the generation of new cells in the striatum. Male mice (n=8) were given METH (30 mg/kg, i.p.) followed by bromo-deoxyuridine (100 mg/kg, i.p.) 36 hours after METH. New cells were visualized by immunostaining with an antibody against bromo-deoxyuridine. There was no bromo-deoxyuridine incorporation in the striatum of mice treated with saline (**A**), however, several bromo-deoxyuridine-positive cells (**B**) are visible throughout all aspects of the striatum. Note at high magnification (100x) the presence of daughter cells that appear to have completed mitotic division (**C**). Bromo-deoxyuridine incorporation is visible in the subventricular zone (SVZ) because this area supports neurogenesis in the adult brain. CC, corpus callosum.

For the proliferation study, data from eight METH and five control animals from each time point were analyzed. For the apoptosis study, seven animals per survival time point were analyzed. Each tissue sample was counted by two different individuals blind to the treatment condition to ensure a minimum 95% correlation in criteria. Proliferating cells were defined as bromo-deoxyuridine-positive nuclei that morphologically appeared to have divided. (see Fig. **1C**). Apoptosis was defined as intensely stained bromo-deoxyuridine-positive round or crescent shaped clumps of compact nuclei indicative of karyopyknosis.

Mean number of nuclei and striatal volumes were quantified using unbiased stereology powered by Stereologer^TM^ software/hardware system from Stereology Resource Center (Chester, MD), connected to a Leica DM 2500 microscope from Leica Microsystems Inc. (Wetzlar, Germany) and a MAC-pro computer system. A cross section of the striatum was outlined at 5X magnification for each tissue sample (see Fig. **1A**). Stereologer’s dissector probe was used for counting and calculating the mean number of nuclei meeting mitotic or pyknotic criteria that fell within the inclusion lines of the optical dissector at 100X magnification. Guard volume was taken as the first and last two micrometers of tissue in cross section. Cells within the guard volume were excluded from the analysis. Statistical analysis was done using Prism software (GraphPad inc. La Jolla, CA). For proliferation, a two-way ANOVA was done to compare mean bromo-deoxyuridine-positive nuclei for each treatment condition at each time point. Post-hoc analysis included Bonferroni multiple comparisons test of significant differences. For pyknosis, the percent difference of bromo-deoxyuridine-positive nuclei at 1.5 days post-METH and bromo-deoxyuridine-positive nuclei that were pyknotic at each time point was calculated and a one-way ANOVA with Dunnet’s multiple comparisons test of differences between 1.5 days post-METH and each of the other time points. The same type of analysis was performed for striatal volume except that the percent difference for each post-METH time point was calculated and compared to saline controls at 1.5 days.

## RESULTS

In previous studies we showed that METH (30 mg/kg, ip) induced the apoptosis of approximately 25% of striatal neurons 24 hours after a single bolus injection [17, 19]. In order to investigate the possibility of production of new cells in the aftermath of the striatal cell loss induced by METH, we injected mice (n=8 per group) with METH (30 mg/kg, i.p.) followed by an injection of bromo-deoxyuridine (100 mg/kg, i.p.) at various times after METH. Bromo-deoxyuridine is a DNA precursor that becomes incorporated into nascent DNA chains in place of thymidine during DNA replication prior to mitotic division. The cells labeled with bromo-deoxyuridine (new cells) can be reliably and conveniently visualized with immunohistochemical methods. Fig. (**1**) shows the emergence of many new cells throughout the striatum at 36 hours after METH (Fig. (**1B**)). Control animals injected with saline display bromo-deoxyuridine-positive cells only in the subventricular zone (Fig. (**1A**)), an area of the adult brain that supports neurogenesis. The new cells shown in Fig. (**1B**) do not migrate to the striatum from the subventricular zone. We base this hypothesis on the observation that pairs of newly divided daughter cells can be found throughout all aspects of the striatum (Fig. (**1C**)).

In order to determine the time(s) after METH when new cells are generated in the striatum, we gave mice bromo-deoxyuridine at various times after METH. In Fig. (**2**) is shown a time course of bromo-deoxyuridine-positive nuclei at various times after a bolus injection of METH. There is a narrow window between 24 and 48 hours after METH when new cells emerge throughout the striatum (Fig. (**2**)). The newly generated cells are not stable and begin to disappear at 2.5 days after METH, or 24 hours after birth (Fig. (**3**)). The bulk of the newly generated cells die by a mechanism of apoptosis displaying pyknotic morphology in their nuclei (data not shown). The new cells continue to die for a period of 12 weeks at which time the majority of the cells (approximately 60%) have disappeared (Fig. (**3**)). At the time when the new cells appear and for most of the 12-week period of protracted cell death the striatum displays an enlargement due to an increase in volume as determined by automated unbiased stereology (Fig. (**4**)). The enlargement of the striatum is largest at one week post-METH because two animals displayed abnormally large striata relative to controls (Fig. (**4**)). The percent increases at 2.5 days and 2 and 4 weeks after METH in Fig. (**4**) did not approach statistical significance at the 95% confidence level. Interestingly, the enlarged striatal morphology normalizes over time (Fig. (**4**)).

## DISCUSSION

The results presented in this study demonstrate that exposure to a single high dose of METH induces the production of new cells (cytogenesis) in the striatum of mice at 24-48 hours after METH. The emergence of new cells is associated with enlarged striatal volume. The newly generated cells are unstable and begin to die from one day after birth up to three months, a phase we refer to as protracted cell death. Most of the newly generated cells die by an apoptotic mechanism associated with pyknotic morphology of the nuclei. The protracted cell death induced by METH is different from the initial phase of cell loss occurring within the first 24 hours after METH. This initial phase involves the loss of approximately 25% of striatal neurons: projection neurons and parvalbumin and cholinergic interneurons. The somatostatin/NPY/NOS interneurons are resistant to METH [19]. Other laboratories have also reported the METH-induced loss of some striatal neurons using TUNEL [15] or Fluoro-Jade C [20]. The protracted phase of striatal cell death induced by METH is restricted to the newly generated cells most of which express the phenotype of glial cells (data not shown). Work in progress in this laboratory is characterizing the phenotype and survival of the newly generated cells in the aftermath of METH.

**Fig. (3):**
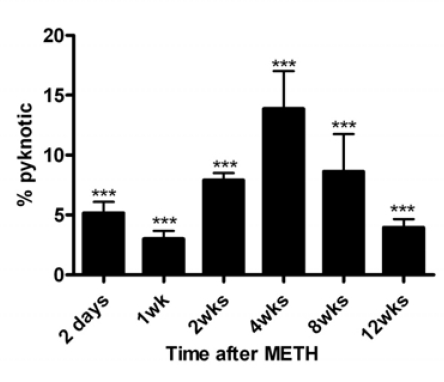
The majority of the newly generated cells die over a three-month period by apoptosis. Mice were treated as in Fig. (**2**) above and the number of pyknotic nuclei (apoptosis) were counted at various times using unbiased stereology starting at 2.5 days after METH. Pyknotic nuclei with bromo-deoxyuridine immunostaining were counted throughout the striatum. Note that the some newly generated cells display the pyknotic phenotype 24 hours after birth, or 2.5 days after METH (bromo-deoxyuridine was injected 1.5 days post-METH). One-way ANOVA showed significant differences between the percent of bromo-deoxyuridine-positive nuclei with pyknotic morphology at various time points after METH in comparison to the 1.5 days time point. ***P<0.0001.

**Fig. (4):**
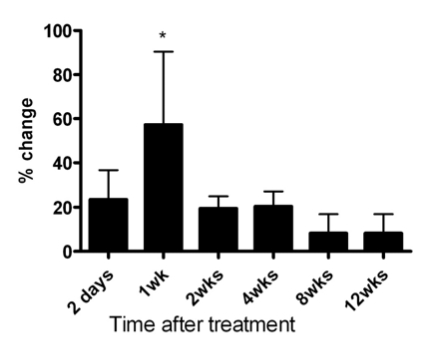
METH induces an increase in striatal volume that normalizes over time. Striatal volume was determined using automated unbiased stereology at the times indicated under the x-axis after a bolus injection of METH (30 mg/kg, i.p.). Note that striatal volumes decrease to near control levels at 8 and 12 weeks post-METH. *p<0.05.

Our results demonstrate that the production of new cells is associated with enlarged striatal morphology. This observation is particularly significant in the light of recent studies showing enlarged striatal volumes in METH users [9, 10, 21]. One study reported that subjects with enlarged putamen and globus pallidus had relatively normal cognitive performance compared to subjects with smaller striatal structures suggesting a compensatory response [9]. Our data demonstrate that the enlarged striatal volume normalizes over a period of three months in mice, the same amount of time associated with the bulk of the protracted cell death (see Figs. **3** & **4**). The human study that found enlarged striatal volumes [9] involved METH users. However, a study assessing abstinent METH users drug-free for approximately two years reported normal striatal volumes [22]. Our results show that the mouse striatum normalizes over a period of three months as most of the newly generated cells die by apoptosis, suggesting that the enlarged striatal volumes observed in METH users may be accounted for by inflammation and the production of new cells.

In conclusion, a bolus high dose of METH induces the genesis of cells in the striatum immediately following the loss of striatal neurons. The production of new cells is associated with enlarged striatal volume that normalizes over a period of three months as the new cells die by apoptosis. A similar pattern of striatal enlargement has been reported with human METH use and normalization after protracted abstinence. More work is needed to determine the role played by the new cells in the aftermath of METH in the striatum.

## Figures and Tables

**Fig. (2):**
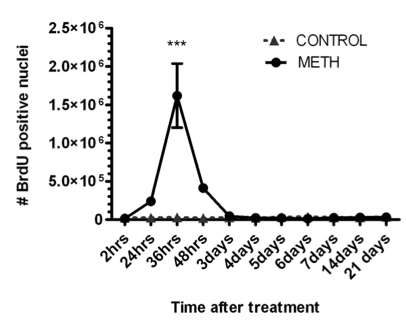
Time course of incorporation of bromo-deoxyuridine into new cells of the striatum after treatment with METH (0 time point). Mice (n=8) were injected with METH (30 mg/kg, i.p.) and were given an injection of bromo-deoxyuridine (100 mg/kg, i.p.) at the times indicated under the x-axis. Sections of striatal tissue were processed for immunohistochemistry with an antibody against bromo-deoxyuridine. Cells were counted throughout the striatum by automated unbiased stereology. Note the 24-hour window between 24 and 48 hours in which robust proliferation of cells occurs in the striatum after METH treatment. Data were analyzed by two-way ANOVA for comparison between the mean number of nuclei with bromo-deoxyuridine incorporation per condition per time point. ***p<0.0001.
